# Supplementary material for: Bioenergetic trade-offs can reveal the path to superior microbial CO2 fixation pathways
Source: mSystems. 2025 Jan 27;10(2):e01274-24. doi: 10.1128/msystems.01274-24 (PMC11834467; doi:10.1128/msystems.01274-24)
Supplement: Supplemental Material — Pathway details, Tables S1 to S3, and Fig. S1 to S3. [file msystems.01274-24-s0001.pdf]

SUPPLEMENTARY MATERIAL

Bioenergetic trade-offs can reveal the path to superior microbial CO_2 fixation pathways

Ahmed Taha, Mauricio Patón and Jorge Rodríguez*

Department of Chemical Engineering, Research and Innovation Centre on CO_2 and H_2 (RICH). Khalifa University. PO Box 127788, Abu Dhabi, United Arab Emirates

*Correspondence: *jorge.rodriguez@ku.ac.ae*; Tel.: +971 2 312 4250

S1. Prokaryotic carbon fixation pathways

Reverse tricarboxylic acid cycle (rTCA)

The rTCA cycle was the first alternative carbon fixing pathway to be discovered (other than the photosynthetic Calvin cycle), dating back to 1966 [1]. The details of the pathway required several decades to be fully studied and acknowledged by microbiologists [2]. The pathway consists of mostly the same reaction steps as those in the widely-known oxidative TCA cycle component of aerobic respiration but proceeding in the reverse direction. In fact, studies have shown that the rTCA cycle has likely evolved first, with the oxidative TCA cycle appearing as an aerobic variation [3]. The direction of several reactions in the rTCA cycle is not spontaneous under standard conditions, and the overall transformation is usually endergonic under typical cellular conditions. Some researchers suggested that the rTCA cycle is the oldest prokaryotic pathway for fixing carbon, and even suggested that a primitive non-enzymatic form of the cycle predates life itself [4]. The pathway has been found to be distributed amongst several organisms in different niches, mainly from the bacterial domain, but examples from the archaeal domain have also been reported [2,5]. The ATP and reducing equivalents are supplied by various catabolic processes in the reported species such as iron oxidation [6], nitrite oxidation [7], hydrogen oxidation [8] or even photosynthesis [5]. Fittingly for such a pathway, only two molecules of ATP are consumed via SLP in the production of one molecule of acetyl-CoA.

The pathway itself consists of two sequences of reactions: the first half (the 'carboxylating arm') is a sequence of reactions where succinyl-CoA is augmented by the addition of two CO_2 molecules and two reducing equivalents into citric acid. The citric acid is then cleaved into acetyl CoA and oxaloacetate (OAA). This part of the cycle is more endergonic, and is also known to be kinetically rate limiting as carboxylases are amongst the slower enzymes [5]. In the second part of the cycle, the OAA is gradually reduced into succinate, which is then activated with free CoA and a substrate-level phosphorylation (SLP) into succinyl-CoA to

restart the cycle. The first carbon fixing reaction is catalyzed by 2-ketoglutarate synthase, an enzyme which is known to be highly sensitive to oxygen [9]. As such, the rTCA is considered to be an anaerobic (or at least, microaerophilic) pathway.

Dicarboxylate hydroxybutyrate cycle (DCHB)

In contrast to the rTCA cycle, the DCHB cycle was the last of the six pathways to be fully elucidated by researchers in 2008 [10]. This was historically a result of this pathway sharing almost all of its reactions with either the rTCA cycle or the 3HP-4HB cycle, making it difficult to isolate and distinguish from the others. Like the rTCA cycle, the pathway is only found in strictly anaerobic microbes. This is a result of the oxygen sensitivity of the pyruvate synthase enzyme employed in the pathway [5]. Unlike the rTCA cycle, the species utilizing this pathway are all from the archaeal domain [11]. Species utilizing the DCHB cycle, such as *Pyrolobus fumarii*, are usually extremely thermophilic and are known to survive at the upper limits of recorded temperature in the range of 90 to 120 ^{o}C [12]. They are also chemolithoautotrophs, generating ATP and reducing equivalents by the oxidation of hydrogen via nitrate or thiosulphate [12].

Like the other cyclic pathways, the DCHB cycle can be divided into a carboxylating arm and a regeneration arm. In the carboxylating arm, one molecule of acetyl CoA is joined with two molecules of CO_2 successively into pyruvate (3C) and OAA (4C). The OAA is then converted into succinyl-CoA using the exact same reaction as the rTCA cycle. The succinyl-CoA is then converted into acetoacetyl-CoA in the regeneration arm, using a series of reactions that involve both reduction and oxidation steps. The acetoacetyl-CoA is then split into two molecules of acetyl-CoA, thus producing the final product and regenerating the cycle precursor in one step. The overall cycle consumes 3.5 ATP equivalents through SLP (fractional values arise because ATP hydrolysis into AMP is considered worth 1.5 ATP equivalents according to [13]).

3-Hydroxypropionate/4-Hydroxybutyrate cycle (3HP-4HB)

The 3HP-4HB cycle is another carbon fixing pathway that was elucidated in the early 21st century by the works of Berg et al [10]. Like the DCHB cycle, this pathway is found only in the archaea domain of life, suggesting a very distinct evolution from bacterial pathways such as the rTCA cycle and 3HP bicycle [14]. However, the 3HP-4HB pathway is utilized by aerobic organisms as all of its enzymes are highly tolerant towards oxygen. This represents an evolutionary advantage for this pathway as the Earth atmosphere grew to be richer in oxygen.

Species utilizing this pathway are also chemolithoautrophs, and are also known to be extremophiles, especially with regards with pH [5,15]. For example, members the archaeal order Sulfolobales utilize this pathway for their carbon fixation needs, and grow at an optimum pH of 2.0, and at temperatures ranging from 60 to 90 ^{o} C [14,16]. They generate ATP and reducing equivalents from the catabolism of hydrogen, iron or sulphur compounds [16].

Similar to the other cyclic pathways, this cycle can be described by a carboxylating arm and a regeneration arm. The regeneration arm of the 3HP-4HB cycle is completely identical to the one in the DCHB cycle. This comes as no surprise as both pathways are achaeal, and none of the enzymes in this part are oxygen sensitive. However, the carboxylating arm is very different; it involves the addition of a first CO_2 molecule to acetyl-CoA at a terminal carbon to generate malonyl-CoA (3C). Three reducing equivalents are then added to reduce malonyl-CoA to propionyl-CoA. A second CO_2 is then added mid-carbon chain, and a small series of reaction steps produce succinyl CoA which enters the regeneration step. The cycle consumes a large amount of ATP equivalents through SLP; five ATP equivalents are spent to fix two CO_2 molecules into acetyl-CoA, making it the most expensive pathway per CO_2 molecule.

3-Hydroxypropionate bicycle (3HP bicycle)

The 3HP bicycle is another aerobic carbon-fixing pathway that was discovered at the turn of the century [17]. The pathway was initially proposed as a cycle that assimilated two CO_2 molecules into glyoxylate in 1993 [18], and the pathway is still referred to as the 3HP cycle in some of the older works. However, since glyoxylate is not one of the central metabolites for building other biomass molecules, further research was conducted until a second cycle that assimilates glyoxylate was completely clarified; thus, the cycle became a bicycle [11]. The pathway has been found exclusively in prokaryotes from the bacterial domain [11], particularly members of the *Chloroflexus* genus [5]. Uniquely amongst the pathways in this work, the one of the enzmes of the 3HP bicycle (methylmalonyl-CoA mutase) is light sensitive, making it functional only in dark environments [11]. This partially explains why this aerobic pathway did not overtake the ubiquitous calvin cycle as the flagship carbon fixation metabolic pathway. Being a bicycle, this pathway involves the largest number of reaction steps amongst all the pathways. Many significant metabolites appear as intermediates in this pathway, making at ideal for metabolising several substrates. In fact, the pathway is frequently utilized in both autotrophic and heterotrophic modes [2]. However, being a longer pathway also inherently hinders its overall rate; this is offset by a rather large amount of ATP expenditure (6 ATP equivalents per bicycle) that serves to create a larger driving force.

Unlike the other pathways in this work, the final product of this pathway is distinctly pyruvate. The first cycle involves the carboxylation and reduction of two molecules of acetyl-CoA into propionyl-CoA. The steps are exactly identical to the steps in the 3HP-4HB cycle. However, all the involved enzymes are very different between the pathways, perhaps due to this pathway being bacterial while the 3HP-4HB cycle being archaeal. This is one of the most interesting examples of two evolutionary lines arriving at the same answer completely independently of each other [2,19]. One of propionyl-CoA molecules continues on the same reaction steps into becoming succinyl-CoA, which is subsequently oxidized into malyl-CoA (5C) which splits into acetyl-CoA and glyoxylate. The acetyl-CoA is ready to restart the first cycle, while the glyoxylate enters the second cycle, merging with the second propionyl CoA into methylmalyl-CoA (5C). After a series of rearrangement steps, the methylmalyl-CoA is split into the final pyruvate product and another acetyl-CoA, thus completely regenerating the bicycle precursors.

Wood Ljungdahl Pathways (WL)

The WL pathways, also known as the reductive acetyl-CoA pathways, are the only non-cyclic pathways known to fix carbon dioxide, although they still involve the cycling of some conserved carriers. The pathways are named after Harland G. Wood and Lars G. Ljungdahl who first discovered the pathway in 1969 [20]. There are two distinct WL pathways: the first occurs in acetogenic bacteria and is utilized both autrotrophically and heterotrophically, while the second exists in methanogenic archaea and is exclusively autotrophic [21]. Since this is the only pathway known to exist in both prokaryotic domains, it is also thought to be the most ancient carbon fixing pathway [2] (in a competing theory to the one mentioned earlier that suggests the rTCA is the oldest one). In addition, this pathway is the one that operates the closest to the thermodynamic limit, with the acetogenic version only requiring one SLP per acetyl-CoA synthesized and the methanogenic version requiring none at all and instead providing the energy entirely through chemiosmotic proton translocations [22]. The WL pathways are anaerobic as their key enzyme, carbon monoxide dehydrogenase, is sensitive to oxygen [11]. Hydrogen is a common terminal electron donor for WL pathways (in acetogens and methanogens) [21], though alternative reported catabolism include sulphate reduction and anaerobic ammonium oxidation (anammox) [2,23]. Still, the WL pathways are limited to quite few niches in nature due to it requiring a large amount of enzyme cofactors, and the high heavy metal requirement in the synthesis of those coenzymes as well as the carbon monoxide dehydrogenase key enzyme [2,11].

WL pathways consist of two linear branches: in the carbonyl branch, CO_2 is reduced to CO using ferredoxin. In the methyl branch, CO_2 is sequentially reduced into formate ($HCOO^-$) and then into methyl (CH_3-) while attached to a cofactor. The cofactor is tetrahydrofolate (THF) in acetogenic WL, and tetrahydromethanopterin (MPT) in methanogenic WL [24]. In the final step, the products of both branches merge into one molecule of acetyl-CoA.

S2. Characteristics of the pathways reactions

The following reaction stoichiometries, possibility of proton translocation and permissible electron carriers have been assembled using the works cited in S1, and biochemical databases [25,26]. Note that the molar ratio of the electron carrier regeneration reactions varies depending on the variant being evaluated.

Table S1. Reaction and biochemical information for propionate oxidation pathways. The numbering of the pathways is in the order presented in S1. Note that the molar ratios for the electron carrier reactions will be negative, indicating that they will proceed in the opposite direction to the one listed in the table

Reaction	Molar Ratio						SLP	Proton translocations permitted?	Possible electron carriers
	P1	P2	P3	P4	P5	P6			
CO_2 Transport	2	2	2	3	2	2	0	Yes	
Succinyl CoA -> 2-ketoglutarate	1	0	0	0	0	0	0	No	NAD, NADP, Fd
2-ketoglutarate -> Isocitrate	1	0	0	0	0	0	0	Yes	NAD, NADP
Isocitrate -> Aconitate	1	0	0	0	0	0	0	No	
Aconitate -> Citrate	1	0	0	0	0	0	0	No	
Citrate -> Oxaloacetate + Acetyl CoA	1	0	0	0	0	0	-1	No	
Oxaloacetate -> Malate	1	1	0	0	0	0	0	Yes	NAD, NADP, Quinone
Malate -> Fumarate	1	1	0	-1	0	0	0	No	
Fumarate -> Succinate	1	1	0	-1	0	0	0	Yes	NAD, FAD, Quinone
Succinate -> Succinyl CoA	1	1	0	0	0	0	-1	No	
Ac-CoA -> Pyruvate	0	1	0	0	0	0	0	No	NAD, NADP, Fd
Pyruvate -> PEP	0	1	0	0	0	0	-1	No	
PEP -> Oxaloacetate	0	1	0	0	0	0	0	No	
Succinyl CoA -> Succinate semialdehyde	0	1	1	0	0	0	0	No	NADP, Fd
Succinate semialdehyde -> 4-hydroxybutyrate	0	1	1	0	0	0	0	No	NAD, NADP
4-hydroxybutyrate -> 4-hydroxybutyryl CoA	0	1	1	0	0	0	-1.5	No	
4-hydroxybutyryl CoA -> Crotonyl CoA	0	1	1	0	0	0	0	No	
Crotonyl CoA -> 3-hydroxybutyryl CoA	0	1	1	0	0	0	0	No	

Table S1. (continued)

Reaction	Molar Ratio						SLP	Proton translocations permitted?	Reported electron carriers
	P1	P2	P3	P4	P5	P6			
3-hydroxybutyryl CoA -> Acetoacetyl CoA	0	1	1	0	0	0	0	No	NAD, NADP
Acetoacetyl CoA -> 2 Acetyl CoA	0	1	1	0	0	0	0	No	
Acetyl CoA -> Malonyl CoA	0	0	1	2	0	0	-1	No	
Malonyl CoA -> Malonate semialdehyde	0	0	1	2	0	0	0	No	NADP
Malonate semialdehyde -> 3-hydroxypropionate	0	0	1	2	0	0	0	No	NAD, NADP
3-hydroxypropionate -> 3-hydroxypropionyl CoA	0	0	1	2	0	0	-1.5	No	
3-hydroxypropionyl CoA -> Acrylyl CoA	0	0	1	2	0	0	0	No	
Acrylyl CoA -> Propionyl CoA	0	0	1	2	0	0	0	Yes	NAD, NADP
Propionyl CoA -> Methymalonyl CoA	0	0	1	1	0	0	-1	No	
Methylmalonyl CoA -> Succinyl CoA	0	0	1	1	0	0	0	No	
Succinyl CoA -> Malyl CoA	0	0	0	1	0	0	0	No	
Malyl CoA -> Acetyl CoA + Glyoxylate	0	0	0	1	0	0	0	No	
Glyoxylate + Propionyl CoA -> Methylmalyl CoA	0	0	0	1	0	0	0	No	
Methylmalyl CoA -> Mesaconyl C1 CoA	0	0	0	1	0	0	0	No	
Mesaconyl C1 CoA -> Mesaconyl C4 CoA	0	0	0	1	0	0	0	No	
Mesaconyl C4 CoA -> Citramalyl CoA	0	0	0	1	0	0	0	No	
Citramalyl CoA -> Pyruvate + Acetyl CoA	0	0	0	1	0	0	0	No	
CO2 -> CO	0	0	0	0	1	1	0	Yes	Fd
CO2 -> Formyl MFR	0	0	0	0	1	0	0	Yes	Fd
Formyl MFR -> Formyl H4MPT	0	0	0	0	1	0	0	No	
Formyl H4MPT -> Methenyl H4MPT	0	0	0	0	1	0	0	No	
Methenyl H4MPT -> Methylene H4MPT	0	0	0	0	1	0	0	No	
Methylene H4MPT -> Methyl H4MPT	0	0	0	0	1	0	0	No	
Methyl H4MPT -> Acetyl CoA	0	0	0	0	1	0	0	No	
Acetyl CoA -> Acetyl Phosphate	0	0	0	0	0	0	0	No	
CO2 -> Formate	0	0	0	0	0	1	0	No	NAD, NADP
Formate +THF -> Formyl THF	0	0	0	0	0	1	-1	No	
Formyl THF -> Methenyl THF	0	0	0	0	0	1	0	No	
Methenyl THF -> Methylene THF	0	0	0	0	0	1	0	No	NAD, NADP
Methylene THF -> Methyl THF	0	0	0	0	0	1	0	Yes	NAD, NADP, Fd
Methyl THF -> Acetyl CoA	0	0	0	0	0	1	0	No	
Acetyl CoA dilution	0	0	0	0	0	0	0	Yes	
F420 (ox) -> F420 (red)	0	0	0	0	2	0	0	Yes	
FADH2 + NAD+ -> FAD+ + NADH							0	Yes	
UQred + NAD+ -> UQox + NADH2							0	Yes	
Fdred -> Fdox							0	Yes	
NADH -> NAD+ + H_2							0	Yes	
NADPH -> NADP+ + H_2							0	Yes	

S3. Environmental, biochemical and thermodynamic parameters

The complete list of standard Gibbs energies of formation used for all the species involved in the pathways are shown in Table S3. Data was assembled from several of sources [24,27–31].

Table S2. Standard Gibbs energies of formation used for all the species involved in the pathways. Some species had to be defined as zero and only the Gibbs energy differences of the reaction used for convenience or data unavailability.

Species Name	ΔG_f^0 (kJ/mol)
Succinyl CoA 1-	-451.42
2-ketoglutarate	-793.41
Coenzyme A	0 (def.)
iso-Citrate 3-	-1156
cis-Aconitate	-917.13
Citrate 3-	-1162.7
Acetyl CoA	-139.42
Acetyl CoA (end)	-139.42
Oxaloacetate	-793.29
Phosphate	-1018.8
Malate 2-	-842.66
Fumarate 2-	-601.87
Succinate 2-	-690.44
Pyruvate	-472.27
Phosphoenolpyruvate 3-	-1263.7
Succinate semialdehyde	-460.24
4-hydroxybutyrate	-501.24
4-Hydroxybutyryl CoA	-275.45
Crotonyl CoA	-47.63
3-Hydroxybutyryl CoA	-289.45
Acetoacetyl CoA	-237.49
Malonyl CoA 1-	-462.01
Malonate semialdehyde	-477.6
3-hydroxypropionate	-518.4
Hydroxypropionyl-CoA	-285
Acryloyl CoA	-46.62
Propionyl CoA	-125.13
Methylmalonyl CoA 1-	-443.22
Malyl CoA -1	-619.82
Glyoxylate	-468.6
Methylmalyl CoA -1	-604.54
Mesaconyl C1 CoA -1	-370.02
Mesaconyl C4 CoA -1	-370.02
Water	-237.18

Species Name	ΔG_f^0 (kJ/mol)
Citramalyl CoA -1	-608.64
Methanofuran	0 (def.)
Formyl methanofuran	-141.22
Tetrahydromethanopterin	0 (def.)
Formyl Tetrahydromethanopterin	-144.72
Methenyl Tetrahydromethanopterin	48.3263
Methylene Tetrahydromethanopterin	78.26
Methyl Tetrahydromethanopterin	64.26
F420	0 (def.)
F420H2	-12.3
Acetyl-Phosphate	-1219.5
Formate	-351
THF	0 (def.)
10-formyl-THF	-131.75
5,10-methenyl-THF	71.4925
5,10-methylene-THF	88.4263
5-methyl-THF	47.4263
Acetate	-369.41
Ethanol	-181.75
Menaquinone (ox)	0 (def.)
Menaquinone (red)	-64.43
Ferredoxin (ox)	0 (def.)
Ferredoxin (red)	79.1177
FAD	0 (def.)
FADH2	-37.4
NAD+	0 (def.)
NADH	21.83
NADP+	0 (def.)
NADPH	21.83
Hydrogen	17.55
CO (aq)	-119.9
Carbon Dioxide	-386
Proton	0

Table S3 shows the default constant environmental and biochemical parameters used for all simulation cases shown unless otherwise stated.

Table S3. Environmental and biochemical parameters for all simulations

Quantity	Value	Unit
Temperature	298	K
Internal pH	7	[]
External pH	7	[]
ΔG_{ATP}	-50	kJ/mol
$r_{H+/ATP}$	10/3	Protons per ATP
[CoA]	0.001	mol/L

S4. Analysis of optimal electron carrier potentials

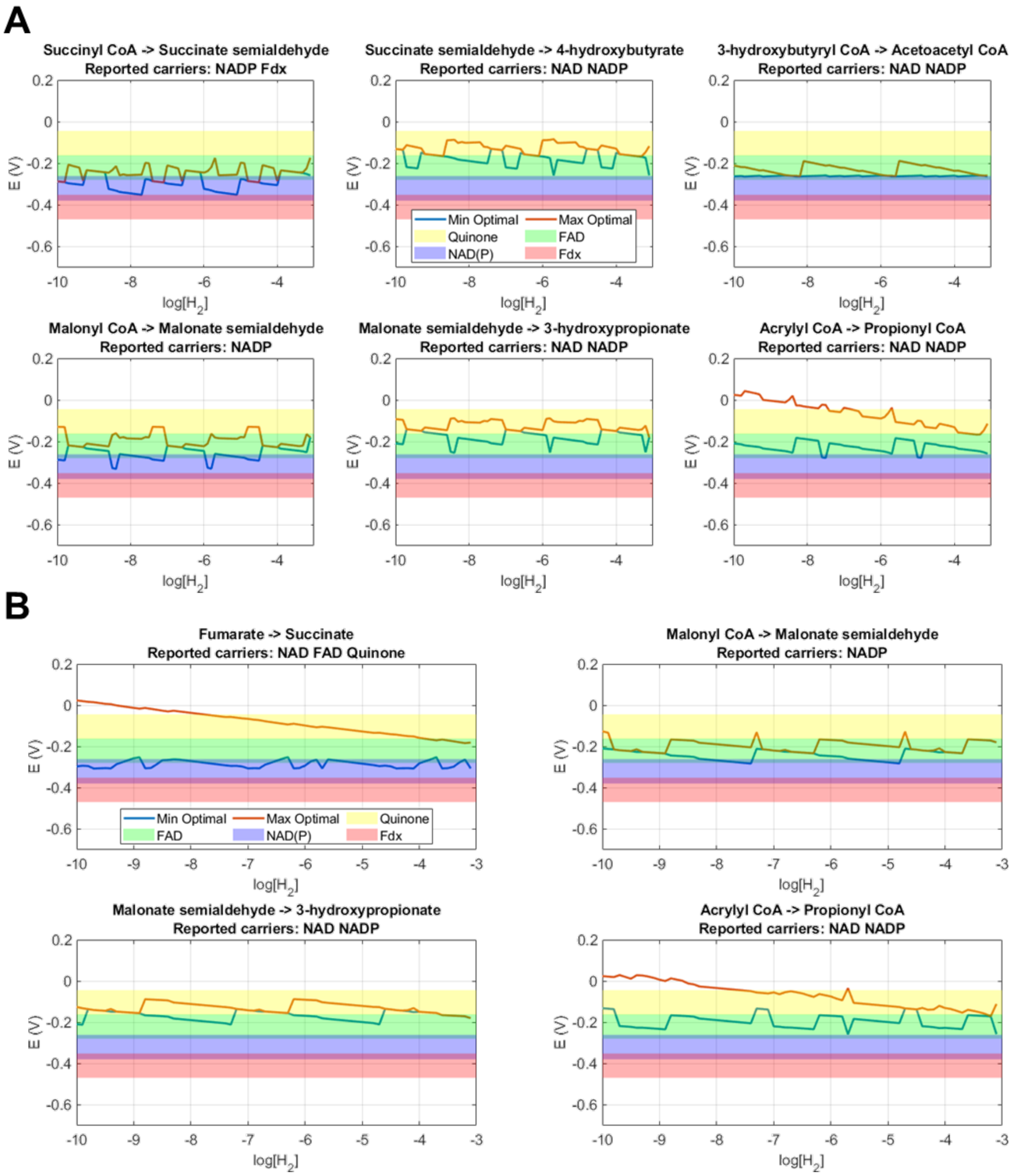

Figure S1: Optimal electron carrier potential ranges for the redox steps in the 3HP-4HB cycle (A) and 3HP bicycle (B) pathways as a function of the electron donor concentration. The shaded ranges represent the potentials of several biochemically known electron carriers (EC) across a concentration ratio ($[EC_{red}]/[EC_{ox}]$) range from 10^{-2} to 10^{2}.

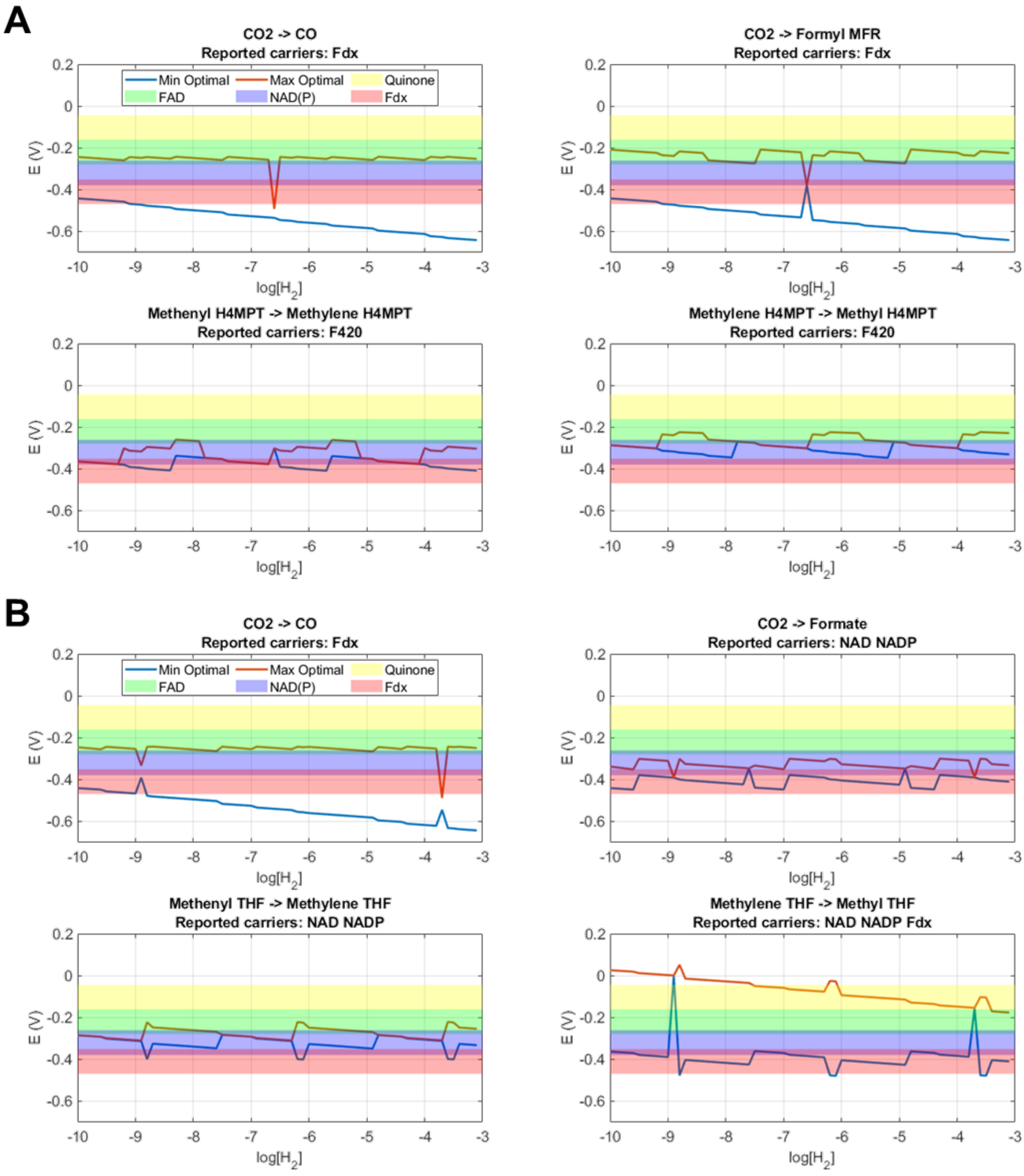

Figure S2: Optimal electron carrier potential ranges for the redox steps in the methanogenic (A) and acetogenic (B) WL pathways as a function of the electron donor concentration. The shaded ranges represent the potentials of several biochemically known electron carriers (EC) across a concentration ratio ($[EC_{red}]/[EC_{ox}]$) range from 10^{-2} to 10^{2}.

S5. Analysis of intermediate metabolite concentrations and pathway bottlenecks

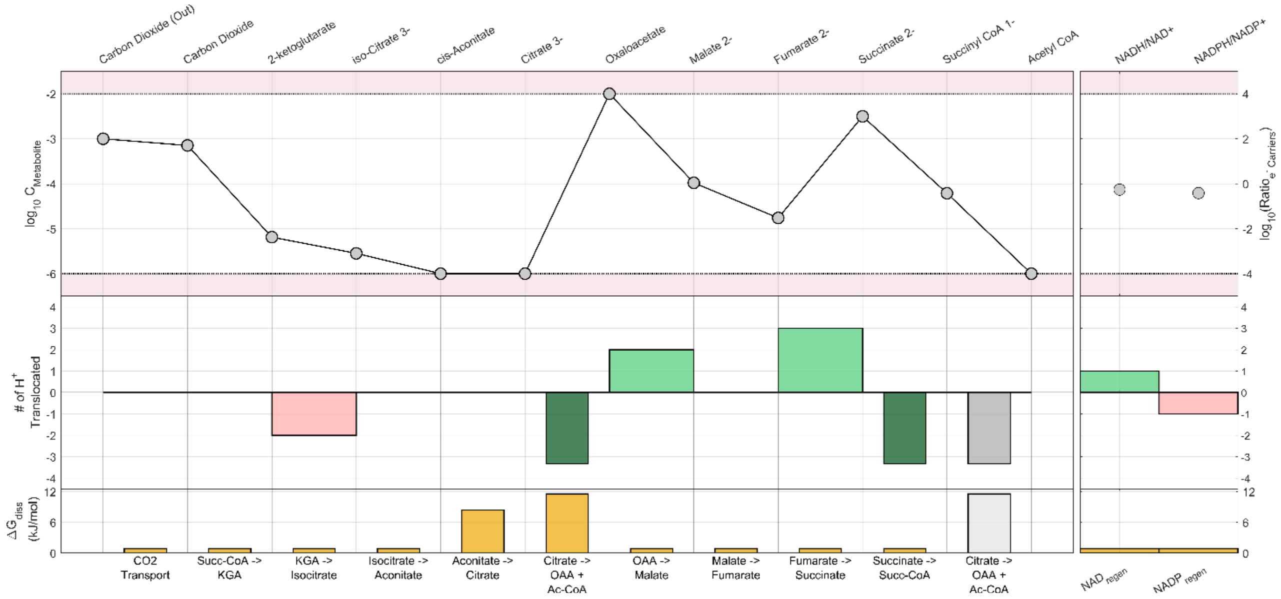

Figure S3. Detailed bioenergetic breakdown of the reverse TCA pathway, evaluated at [CO_2] = 0.001 mol/L. The top part presents the intermediate metabolite concentrations. The species are arranged in the order they react in the pathway, allowing for visual identification of bottlenecks. The wide red bars represent proton translocations invested and the bright green bars represent proton translocations recovered. The narrow dark green bars represent SLP. A greyed-out bar indicates a reaction that is repeated for the sequential listing of species.

References

1. Evans MC, Buchanan BB, Arnon DI. A new ferredoxin-dependent carbon reduction cycle in a photosynthetic bacterium. *Proceedings of the National Academy of Sciences* 1966;**55**:928–34.

2. Hügler M, Sievert SM. Beyond the Calvin cycle: autotrophic carbon fixation in the ocean. *Ann Rev Mar Sci* 2011;**3**:261–89.

3. Aoshima M. Novel enzyme reactions related to the tricarboxylic acid cycle: phylogenetic/functional implications and biotechnological applications. *Appl Microbiol Biotechnol* 2007;**75**:249–55.

4. Wächtershäuser G. Evolution of the first metabolic cycles. *Proceedings of the National Academy of Sciences* 1990;**87**:200–4.

5. Bar-Even A, Noor E, Milo R. A survey of carbon fixation pathways through a quantitative lens. *J Exp Bot* 2012;**63**:2325–42.

6. Levicán G, Ugalde JA, Ehrenfeld N *et al.* Comparative genomic analysis of carbon and nitrogen assimilation mechanisms in three indigenous bioleaching bacteria: predictions and validations. *BMC Genomics* 2008;**9**:581.

7. Lücker S, Wagner M, Maixner F *et al.* A Nitrospira metagenome illuminates the physiology and evolution of globally important nitrite-oxidizing bacteria. *Proceedings of the National Academy of Sciences* 2010;**107**:13479–84.

8. Yamamoto M, Ikeda T, Arai H *et al.* Carboxylation reaction catalyzed by 2-oxoglutarate:ferredoxin oxidoreductases from Hydrogenobacter thermophilus. *Extremophiles* 2010;**14**:79–85.

9. Imlay JA. Iron-sulphur clusters and the problem with oxygen. *Mol Microbiol* 2006;**59**:1073–82.

10. Huber H, Gallenberger M, Jahn U *et al.* A dicarboxylate/4-hydroxybutyrate autotrophic carbon assimilation cycle in the hyperthermophilic Archaeum Ignicoccus hospitalis. *Proc Natl Acad Sci U S A* 2008;**105**:7851–6.

11. Berg IA. Ecological Aspects of the Distribution of Different Autotrophic CO2 Fixation Pathways. *Appl Environ Microbiol* 2011;**77**:1925–36.

12. Blöchl E, Rachel R, Burggraf S *et al.* Pyrolobus fumarii, gen. and sp. nov., represents a novel group of archaea, extending the upper temperature limit for life to 113 degrees C. *Extremophiles* 1997;**1**:14–21.

13. Frey PA, Arabshahi A. Standard free energy change for the hydrolysis of the alpha, beta-phosphoanhydride bridge in ATP. *Biochemistry* 1995;**34**:11307–10.

14. Berg IA, Kockelkorn D, Ramos-Vera WH *et al.* Autotrophic carbon fixation in archaea. *Nat Rev Microbiol* 2010;**8**:447–60.

15. Hawkins AS, Han Y, Lian H *et al.* Extremely Thermophilic Routes to Microbial Electrofuels. *ACS Catal* 2011;**1**:1043–50.

16. Auernik KS, Cooper CR, Kelly RM. Life in hot acid: pathway analyses in extremely thermoacidophilic archaea. *Curr Opin Biotechnol* 2008;**19**:445–53.

17. Herter S, Fuchs G, Bacher A *et al.* A Bicyclic Autotrophic CO2 Fixation Pathway in Chloroflexus aurantiacus *. *Journal of Biological Chemistry* 2002;**277**:20277–83.

18. Strauss G, Fuchs G. Enzymes of a novel autotrophic CO2 fixation pathway in the phototrophic bacterium Chloroflexus aurantiacus, the 3-hydroxypropionate cycle. *Eur J Biochem* 1993;**215**:633–43.

19. Fuchs G. Alternative Pathways of Carbon Dioxide Fixation: Insights into the Early Evolution of Life? *Annu Rev Microbiol* 2011;**65**:631–58.

20. Ljungdahl LG, Wood HG. Total Synthesis of Acetate from Co2 by Heterotrophic Bacteria. *Annual Review of Microbiology* 1969;**23**:515–38.

21. Ragsdale SW, Pierce E. Acetogenesis and the Wood-Ljungdahl pathway of CO(2) fixation. *Biochim Biophys Acta* 2008;**1784**:1873–98.

22. Mitchell P. Coupling of phosphorylation to electron and hydrogen transfer by a chemi-osmotic type of mechanism. *Nature* 1961;**191**:144–8.

23. Strous M, Pelletier E, Mangenot S *et al.* Deciphering the evolution and metabolism of an anammox bacterium from a community genome. *Nature* 2006;**440**:790–4.

24. Maden BE. Tetrahydrofolate and tetrahydromethanopterin compared: functionally distinct carriers in C1 metabolism. *Biochem J* 2000;**350**:609–29.

25. Caspi R, Altman T, Billington R *et al.* The MetaCyc database of metabolic pathways and enzymes and the BioCyc collection of Pathway/Genome Databases. *Nucleic Acids Research* 2014;**42**:D459–71.

26. Kanehisa M, Goto S. KEGG: kyoto encyclopedia of genes and genomes. *Nucleic Acids Res* 2000;**28**:27–30.

27. Thauer RK, Jungermann K, Decker K. Energy conservation in chemotrophic anaerobic bacteria. 1977;**41**:81.

28. Hanselmann KW. Microbial energetics applied to waste repositories. *Experientia* 1991;**47**:645–87.

29. Alberty RA. *Biochemical Thermodynamics: Applications of Mathematica*. John Wiley & Sons, 2006.

30. Heijnen J. Bioenergetics of Microbial Growth. In: Flickinger M, Drew S (eds.). *Encyclopedia of Bioprocess Technology: Fermentation, Biocatalysis and Bioseparation*. Chichester UK: John Wiley & Sons, 1999, 267–91.

31. Flamholz A, Noor E, Bar-Even A *et al.* eQuilibrator—the biochemical thermodynamics calculator. *Nucleic Acids Res* 2012;**40**:D770–5.
